# Prevalence, severity, and determinants of CKD-associated pruritus in a Swiss hemodialysis population with widespread use of hemodiafiltration: a cross-sectional study

**DOI:** 10.1186/s12882-025-04570-w

**Published:** 2025-11-17

**Authors:** Nancy Helou, Dina Nobre, Tanguy Corre, Olivier Bonny, Robin Chazot, Anne Cherpillod, Sophie De Seigneux, David Fumeaux, Zina Fumeaux, Antoine Humbert, David Jaques, Ould Maouloud Hemett, Patricia Mehier, Grzegorz Nowak, Olivier Phan, Anne-Hélène Reboux, Alain Rossier, Floriane Seydtaghia, Daniel Teta, Gérard Vogel, Menno Pruijm

**Affiliations:** 1 School of Health Sciences (HESAV), HES-SO University of Applied Sciences and Arts Western Switzerland, Avenue de Beaumont 21, Lausanne, 1011 Switzerland; 2 Service of Nephrology and Hypertension, University Hospital of Lausanne and University of Lausanne (CHUV), Lausanne, Switzerland; 3 Service of Nephrology, Hôpital de Fribourg (HFR) and University of Fribourg, Fribourg, Switzerland; 4 Dialyse Riviera, Vevey, Switzerland; 5 Dialysis Center, Clinique Cecil, Lausanne, Switzerland; 6 Service of Nephrology, Geneva University Hospitals (HUG), Geneva, Switzerland; 7 Dialysis Center, Nyon Hospital, Nyon, Switzerland; 8 Service of Nephrology, Réseau Hospitalier Neuchâtelois, Neuchâtel, Switzerland; 9 Dialysis Center, Hôpital Riviera-Chablais, Rennaz, Switzerland; 10 Dialysis Center, Hôpital du Valais – Centre Hospitalier du Valais Romand (CHVR), Hôpital de Sierre, Sierre, Switzerland; 11 Hemodialysis Center, Broye Intercantonal Hospital, Payerne, Switzerland; 12 Dialysis Center, Hôpital du Valais – Centre Hospitalier du Valais Romand (CHVR), Hôpital de Martigny, Martigny, Switzerland; 13 Dialysis Center, Etablissements Hospitaliers du Nord Vaudois (eHnv), Yverdon-les-Bains, Switzerland; 14 Service of Nephrology, Hôpital du Valais – Centre Hospitalier du Valais Romand (CHVR), Hôpitaux de Martigny, Sierre et Sion, Sion, Switzerland

**Keywords:** Chronic kidney disease-associated pruritus, Chronic hemodialysis, Itch, Itch severity, Patient-reported outcomes

## Abstract

**Background:**

Chronic Kidney Disease Associated-Pruritus (CKD-aP) is frequent among hemodialysis patients. Its associations with hemodiafiltration or conventional hemodialysis and clinical characteristics are inconsistent in observational studies. This study aimed to assess the prevalence, severity and factors associated with CKD-aP among patients on hemodialysis in the French-speaking part of Switzerland, where hemodiafiltration is widely used.

**Methods:**

A cross-sectional design was used. Adults on hemodialysis for ≥ 6 months and free of cognitive impairment were recruited from 15 dialysis centers. A research nurse collected sociodemographic, clinical, laboratory data and assessed CKD-aP using the Visual Analogue Scale (VAS) and Verbal Rating Scale (VRS). A multilevel mixed-effects model with maximum likelihood estimation was performed to investigate associations.

**Results:**

A total of 413 participants, mean age was 69.68 years (SD 13.52), were included; 66% were men, 73% on hemodiafiltration. CKD-aP prevalence was 25%. VAS average itch score of participants with CKD-aP was 4.74 ± 1.99, versus 6.62 ± 2.18 for the worst intensity itch. According to VRS, most participants with CKD-aP suffered moderate (61.4%) to severe (22%) itch within the past 24 h. Higher phosphorus levels were significantly associated with increased CKD-aP severity, but not prevalence. Depression, smoking, and polypharmacy were associated with increased CKD-aP severity, whereas hemodialysis modality, volume substitution, filter or access type were not.

**Conclusions:**

Despite high dialysis standards, CKD-aP remains a frequent symptom in Switzerland. Depression is associated with CKD-aP and its severity while hemodialysis modality is not. These results suggest that effective management of CKD-aP requires not only optimal dialysis prescription but also a holistic approach.

**Trial registration:**

Registered at ClinicalTrials.gov (NCT05524467) on 10.06.2022.

**Supplementary Information:**

The online version contains supplementary material available at 10.1186/s12882-025-04570-w.

## Background

Chronic Kidney Disease-Associated Pruritus (CKD-aP) is a distressing skin itch fequently experienced by patients on dialysis [1]. The Dialysis Outcomes and Practice Patterns Study (DOPPS) reported a prevalence of 42% among patients receiving Hemodialysis (HD) [2], while a meta-analysis found a slightly higher prevalence of 55%, with significant heterogeneity between included studies and notable country-level differences [3]. Differences in CKD-aP prevalence across countries remains unclear, likely reflecting differences in screening methods, patient characteristics, dialysis prescription or modalities and the inconsistent use of validated patient-reported outcome measures [3]. Many studies relied on patients’ extensive self-reported questionnaires, without personal interviews. DOPPS assessed CKD-aP with a single question from the Kidney Disease Quality of Life Short Form KDQOL-SF [2, 4], which is similar to the Verbal Rating Scale (VRS) with five levels severity scoring. Other studies used the Numeric Rating Scale (NRS), the Visual Analogue Scale (VAS), the Itch Severity Scale ISS, or the Itchy QoL [5]. This underscores the need for studies using standardized validated tools to assess CKD-aP prevalence and severity.

CKD-aP is a main concern to patients, as it is associated with sleep disturbances, compromised Quality of Life (QoL) [6–10], depressive symptoms [11], anxiety, increased risk of hospitalizations and death [6–8]. Severe CKD-aP is also linked to decreased engagement in HD [11, 12]. Itch and sleep disturbance are among the symptoms identified as unmet needs by HD patients [13]. A study ranked the needs of patients with CKD-aP and reported that the second most important identified need was “no longer experience itch” [14].

The pathogenesis of CKD-aP is complex and multifactorial. CKD-aP is thought to be related to an increase in circulating uremic toxins, skin microinflammation, systemic inflammation, peripheral neuropathy with neuropathic itchiness, and opioid imbalance [1, 4, 15]. The literature suggests that CKD-aP is induced by a low Kt/V or high calcium-phosphate product, leading to the deposition of uremic toxins and electrolytes in the skin [1, 9]. Despite these assumptions, associations between CKD-aP and clinical characteristics such as serum calcium, phosphorus, albumin, ferritin levels are inconsistent in observational studies [4, 11, 15, 16]. DOPPS phases 4 to 6 data indicated an increased itch score at higher serum phosphorus levels, while DOPPS 5 showed no relationship [4, 11]. KDIGO further notes that the association between mineral metabolism and CKD-aP remains unconfirmed, while emphasizing dialysis optimization as a key strategy to reduce its burden [17].

Most studies predate the recent widespread adoption of Hemodiafiltration (HDF), and it remains unclear whether similar associations and prevalence rates apply to patients receiving HDF.

The variability in CKD-aP prevalence estimates across countries, the unclear role of sociodemographic and clinical determinants, and the limited use of validated assessment tools highlight the need for studies that accurately assess both prevalence and severity using validated measures. Evaluating CKD-aP in Switzerland, within a high-quality care setting characterized by widespread HDF prescription and unrestricted access to therapies, is particularly relevant. Moreover, the expected increase in the dialysis population will likely increase the burden of CKD-aP [18]. The availability of new drugs such as kappa-opioid receptor agonists further emphasizes the importance of precise prevalence estimates to guide clinical decision-making. This study also addresses gaps identified by KDIGO and support patient-centered care for this underrecognized and undertreated condition.

## Methods

### Aim of the study

The aim of this study was to determine the prevalence and severity of CKD-aP in adults on HD in Switzerland and their associations with dialysis modality, as well as various sociodemographic and clinical factors.

### Specific objectives

1. To determine the prevalence and severity of CKD-aP in adults undergoing hemodialysis in Switzerland.
2. To identify associations between hemodialysis-related factors (e.g. HDF versus conventional hemodialysis, dialysis duration, eKt/V, filter type, or substitution volume) and the occurrence and severity of CKD-aP.
3. To identify associations between different sociodemographic variables (e.g. age, sex) and clinical variables (e.g. serum phosphorus, serum creatinine) and the occurrence and severity of CKD-aP.

### Design

A multicentric cross-sectional design was used to determine the prevalence and severity of CKD-aP among patients on chronic HD, in the French-speaking region of Switzerland.

### Setting

The study involved 15 centers, including academic, public hospitals, and private dialysis centers. Recruitment and data collection were conducted between 01.09.2022–09.10.2023, before the availability of Difelikefalin (Kapruvia^®^) in Switzerland, a kappa-opoid receptor KOR agonist indicated for treatment of moderate-to-severe CKD-aP in adult HD patients. The study is reported in accordance with STROBE [19].

### Participants

Participants were recruited by the treating nephrologist and the research nurse, based on the following inclusion criteria: age ≥ 18 years, on HD ≥ 6 months for end-stage kidney disease, and French-speaking. Exclusion criteria included non-ambulatory or hospitalized patients, patients on peritoneal dialysis, or with cognitive impairment. Only CKD-related pruritus was analyzed; pruritus from other causes or underlying skin conditions was excluded.

### Prevalence and severity of CKD-aP

CKD-aP prevalence and severity were measured using a specific question.

1. “Do you have itching?” and the French versions of two questionnaires: 2) VAS and 3) VRS.
2. VAS includes two questions: the first refers to the average itch intensity within the past 24 h and the second refers to the worst itch-numeric rating scale WI-NRS and addresses the worst itch intensity experienced within the past 24 h. VAS scores range from 0 (not itchy) to 10 (extremely itchy, worst itch imaginable).
3. VRS includes two questions: the first refers to the average itch intensity within the past 24 h and the second refers to the worst itch WI-VRS. VRS is a five-point scale and consists of 5 adjectives describing levels of symptom intensity (0 = no itch, 1 = mild itch, 2 = moderate itch, 3 = severe itch and 4 = very severe itch) [20].

The research nurse performed on-site visits and interviews using the VAS and VRS to assess CKD-aP. She checked the skin of each participant for redness and itching signs (scratch marks, prurigo nodularis, scars) and assessed whether there was an alternative underlying diagnosis (skin disease) for pruritus. All participants who reported a VRS or VAS score of 1 or higher were classified as having CKD-aP, in the absence of an alternative skin diagnosis.

### Sociodemographic and clinical variables

Sociodemographic data (age, sex, marital status, etc.) were collected from participants and clinical data (diagnosis, laboratory) from medical records, along with prescribed medications for CKD-aP including antihistamines, gabapentinoids, opioid receptor modulators (Butorphanol, Naltrexin), antidepressants, and emollients. The medication count reflected the number of daily pills prescribed for ≥ 14 consecutive days, whether regular or as needed.

### Data management and statistical analysis

Data were entered into REDCap™ and double checked by the project leader (N.H). Study documents were stored in a secured, password-protected SWITCHdrive folder.

Based on the 42% reported CKD-aP prevalence in the literature [2], a sample size of 375 participants was required to achieve a 5% margin of error with a 95–99% Confidence Interval. Analyses followed a full inclusion approach, whereby all participants were included even if some variables had missing values, to minimize bias due to data exclusion. Sample sizes (n) for each variable are reported in Table 1 to describe data completeness. Missing values were low overall and not systematically distributed across centers nor patient categories. No imputation was performed for missing data. All statistical analyses were performed using STATA version 16. Histograms were used to assess normal distribution. Categorical variables were expressed in % and absolute numbers. Means, medians and standard deviations were computed for continuous numeric variables. Regression analysis showed significant differences in CKD-aP prevalence between participating centers. To account for this variability, we used a multilevel mixed-effects model with Maximum Likelihood Estimation (MLE) for data analysis. This approach includes a random intercept for each center, allowing to model the between-center variability effectively. It improves the precision of fixed-effect estimates by accounting for random effects, thereby reducing the risk of biased parameter estimates [21]. All other variables were included as fixed effects. Variables with a significance level α ≤ 0.05 in the univariate mixed-effects models were then included in the final multiple mixed-effects model with MLE. All variables considered in the analysis were pre-specified in the study protocol based on existing literature and clinical relevance. This approach ensured that both clinical and statistical considerations were incorporated, while reducing the risk of model overfitting. A biostatistician (T.C.) impartially oversaw the data analysis.

## Results

**Fig. 1:**
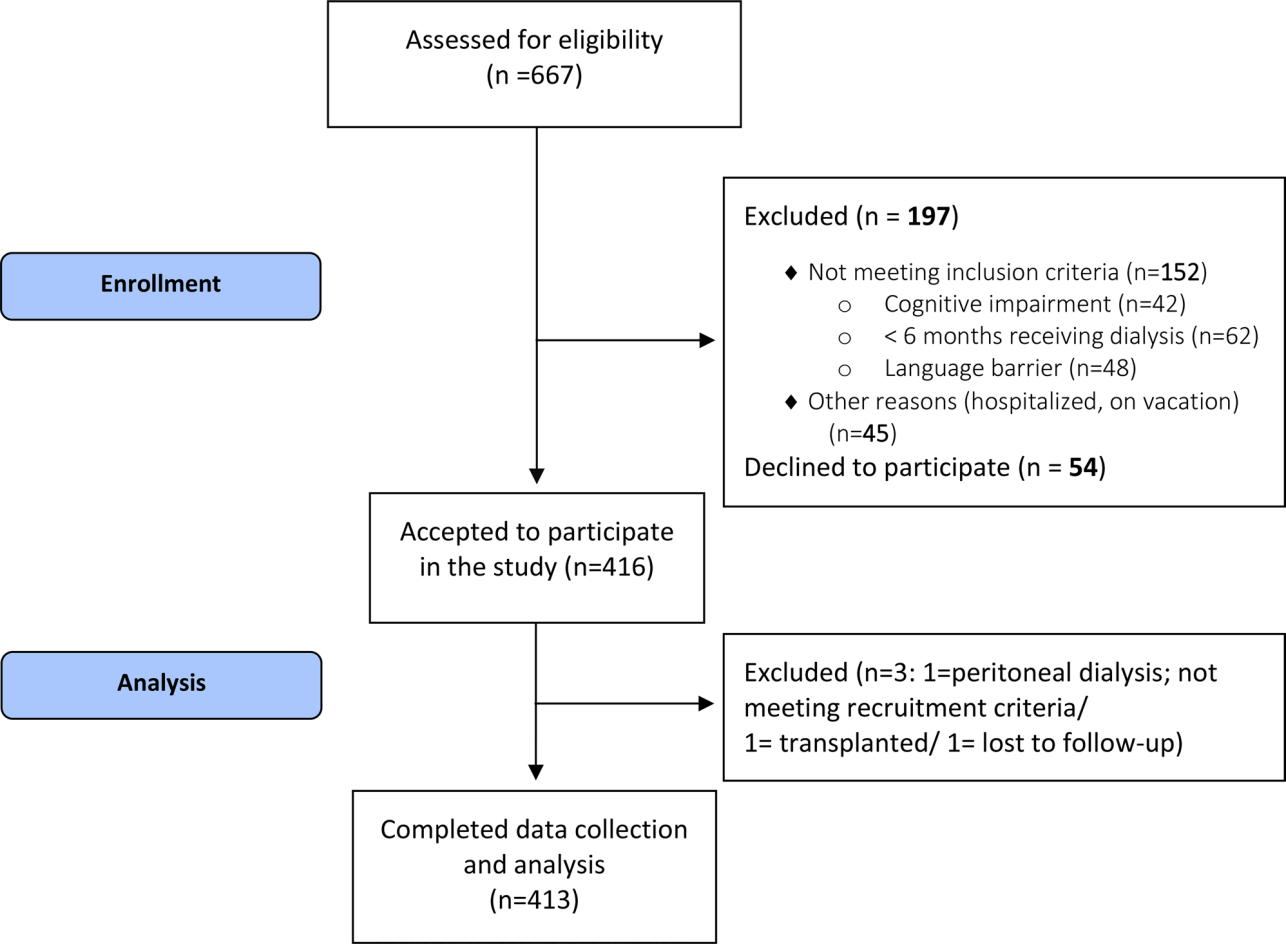
Study profile

413 patients from 15 dialysis centers participated in the study. The participation rate of eligible patients was 88.5% (Fig. 1).

### Patient characteristics

Participants had a mean age of 69.7 years (SD 13.5) and 66% were men. Characteristics of the study population are summarized in Table 1. Eleven participants had alternative skin conditions but did not have CKD-aP. These patients were included in the study. Only 1 participant presented with both CKD-aP and an alternative skin condition.

**Table 1:** Sociodemographic and clinical characteristics of participants

		All participants (*n* = 413)	Participants without CKD-aP (*n* = 312)	Participants with CKD-aP (*n* = 101)
Age, years, mean (SD)		69.68 (13.5)	69.3 (13.8)	70.8 (12.8)
Sex	Men, %	66	66	65
Marital Status	Single, %	16	15	20
	Married, in couple %	52	52	51
	Separated, %	2	2	1
	Divorced, %	14	15	10
	Widowed, %	16	16	18
Professional activity	Employed, %	7	7	5
	Self-employed, %	3	3	5
	Unemployed, %	2	2	3
	Retired, %	70	69	70
	Disability leave, %	18	19	17
Living alone, %		31	31	33
Smoker, %		18	16	25
BMI, kg/m^2^, mean (SD)		26.91 (5.5)	26.6 (5.2)	27.8 (6.5)
# years on dialysis, years, mean (SD; n)		4.20 (5; *n* = 412)	4.1 (5.2; *n* = 311)	4.5 (4.5; *n* = 101)
Dialysis Treatment	Hemodialysis, %	27	28	24
	Hemodiafiltration, %	73	72	76
Volume substitution, L, mean (SD; n)		21.64 (7.08; *n* = 248)	22.06 (7.26; *n* = 187)	20.35 (6.38; *n* = 61)
Vascular access	Fistula, %	64	64	63
	Permcath, %	36	36	37
Dialysis frequency, number/week, mean (SD)		2.94 (0.32)	2.95 (0.34)	2.93 (0.26)
Dialysis session duration, hours, mean (SD)		3.78 (0.36)	3.78 (0.36)	3.79 (0.37)
eKt/V, mean (SD; n)		1.49 (0.33; *n* = 371)	1.49 (0.34; *n* = 275)	1.47 (0.32; *n* = 96)
Diabetes, %		44	43	45
Peripheral Vascular Disease, %		44	43	48
Hypertension, % (n)		85	85	83
Congestive Heart Failure, %		20	18	23
Severe Covid-19 complications, %		4	4	6
Physician-diagnosed depression, %		10	8	17
# prescribed medications, pills/day, mean (SD)		15.29 (4.91)	15.12 (4.94)	15.82 (4.36)
Serum albumin, g/L, mean (SD; n)		38.51 (4.62; *n* = 408)	38.54 (4.48; *n* = 308)	38.42 (5.04; *n* = 100)
Urea, mmol/L, mean (SD)		19.55 (12.46)	19.99 (13.76)	18.17 (6.97)
Residual Urea Clearance (ml/min), mean (SD; n)		5.54 (6.68; *n* = 32)	5.68 (6.27; *n* = 20)	5.29 (7.60; *n* = 12)
CRP, mg/L, mean (SD; n)		18.73 (26.8; *n* = 320)	18.03 (26.23; *n* = 239)	20.79 (28.48; *n* = 81)
Hemoglobin, g/L, mean (SD)		110.86 (12.56)	111.32 (12.1)	109.43 (13.87)
Phosphorus, mmol/L, mean (SD; n)		1.51 (0.42; *n* = 411)	1.51 (0.4; *n* = 310)	1.54 (0.46; *n* = 101)
Calcium, mmol/L, mean (SD; n)		2.23 (0.16; *n* = 412)	2.23 (0.17; *n* = 311)	2.26 (0.15; *n* = 101)
Potassium, mmol/L, mean (SD)		4.83 (0.67)	4.83 (0.67)	4.8 (0.64)
y-GT, U/L, mean (SD; n)		49.51 (69.8; *n* = 370)	45.97 (65.16; *n* = 285)	61.39 (82.83; *n* = 85)
Total Bilirubin, µmol/L¸ mean (SD; n)		7.90 (7.76; *n* = 139)	7.87 (8.48; *n* = 110)	8.01 (4.1; *n* = 29)
Ferritin, µg/L, mean (SD; n)		546.85 (371.27; *n* = 403)	531.05 (322.23; *n* = 303)	594.73 (489.78; *n* = 100)
PTH, pmol/L, mean (SD; n)		32.06 (24.37; *n* = 398)	31.74 (24.48; *n* = 298)	33.01 (24.13; *n* = 100)

### CKD-aP prevalence and CKD-aP severity

**Fig. 2:**
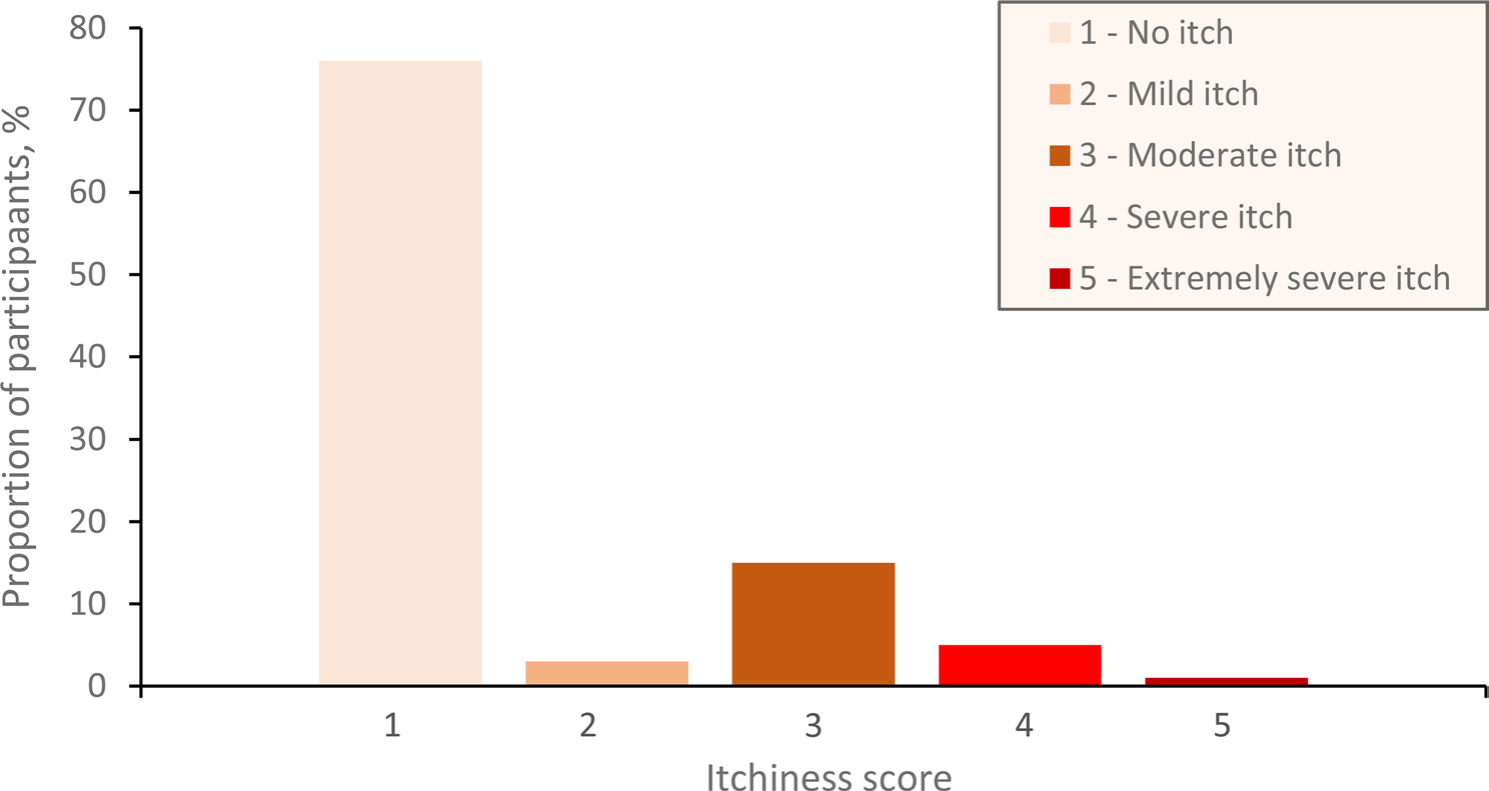
Verbal Rating Scale - VRS average itch score within the past 24 h (*n* = 413)

CKD-aP overall prevalence was 24.5% (Fig. 2), ranging from 8% to 45% across 15 centers. Eight centers had rates between 20 and 33%.

### Characteristics of participants with CKD-aP

Among participants CKD-aP (*n* = 101), 71% reported generalized itch and 35% waking up at night because of the itch. Mean VAS score of participants with CKD-aP was 4.74 (SD 1.99) and 6.62 (SD 2.18) for worst intensity itch, within the past 24 h. Most participants with CKD-aP had moderate (61%) to severe (25%) itch and 71% experienced at least one severe episode within the past 24 h. All participants with CKD-aP (*n* = 101) reported experiencing itching within the past 24 h.

### CKD-aP and itch severity associations with sociodemographic and clinical variables

The likelihood ratio (LR) tests (χ²), comparing the univariate random regression model to the fixed univariate regression models, were significant for all independent variables, indicating that the random regression model is more appropriate for this analysis.

The multilevel mixed-effects model, with a random intercept for centers, revealed significant positive associations between physician-diagnosed depression, CKD-aP, and itch severity for both VAS and VRS scores (Tables 2, 3 and 4). Serum phosphorus was not associated with an increased prevalence of CKD-aP, but among patients with CKD-aP, it was associated with greater itch severity, measured by the VAS for the worst itch within the past 24 h (Table 3). The number of prescribed medications was not associated with increased risk of CKD-aP, but with the severity of the itch in those with CKD-aP (Tables 3 and 4).

**Table 2:** Association between CKD-aP prevalence and clinical variables (*n* = 413)

CKD-aP = Yes	Coef.	SE	95% CI		*p*
			*LL*	*UL*	
(Intercept)	-0.171	0.160	-0.485	0.142	0.284
**Age** (years)	0.002	0.002	-0.001	0.006	0.119
**Sex** Woman	0.008	0.044	-0.078	0.094	0.856
**BMI** (kg/m2)	0.008	0.004	0.001	0.015	0.031
**Physician-diagnosed depression**	0.188	0.070	0.051	0.325	0.007

**Random effects**	Estimate	SE	95% CI		
Dialysis centers			*LL*	*UL*	
Constant Variance	0.005	0.004	0.001	0.022	
Residual Variance	0.174	0.012	0.152	0.200	

Analysis accounted for variations between dialysis centers by using a random intercept in the regression model. Likelihood Ratio (LR) χ² = 4.3, *p* = 0.019. * Significance at *p* ≤ 0.05. Coef. = Coefficient; SE = Standard Error; CI = confidence interval; *LL* = lower limit; *UL* = upper limit

**Table 3:** Association between the VAS itch score and clinical variables (*n* = 413)

VAS average itch within the past 24 h (numeric score 0 no itch-10 worst)	Coef.	SE	95% CI		*p*
			*LL*	*UL*	
(Intercept)	-0.581	0.716	-1.985	0.823	0.417
**Age** (years)	0.008	0.008	-0.008	0.024	0.318
**Sex** Woman	0.177	0.227	-0.268	0.622	0.436
**Physician-diagnosed depression**	1.204	0.360	0.498	1.911	0.001
**Number of prescribed medications**	0.061	0.023	0.016	0.107	0.009

**Random effects**	Estimate	SE	95% CI		
Dialysis centers			*LL*	*UL*	
Constant Variance	0.266	0.155	0.085	0.833	
Residual Variance	4.597	0.325	4.003	5.280	
Likelihood Ratio (LR) χ² = 12.33, *p* = 0.0002					

**VAS worst itch** within the past 24 h (numeric score 0 no itch to 10 worst)	Coef.	*SE*	95% CI		*p*
			*LL*	*UL*	
(Intercept)	-1.900	1.200	-4.252	0.453	0.113
**Age** (years)	0.011	0.011	-0.011	0.033	0.336
**Sex** Woman	0.184	0.306	-0.416	0.783	0.548
**Smoking** Yes	0.911	0.379	0.168	1.653	0.016
**Physician-diagnosed depression**	1.503	0.486	0.550	2.455	0.002
**Number of prescribed medications**	0.074	0.031	0.012	0.135	0.019
**Phosphorus** (mmol/L)	0.807	0.359	0.104	1.511	0.024

**Random effects**	Estimate	SE	95% CI		
Dialysis centers			*LL*	*UL*	
Constant Variance	0.339	0.224	0.093	1.239	
Residual Variance	8.348	0.591	7.267	9.590	
Likelihood Ratio (LR) χ² = 7.19, *p* = 0.0037					

Analysis accounted for variations between dialysis centers by using a random intercept in the regression model. * Significance at *p* ≤ 0.05. Coef. = Coefficient; SE = Standard Error; CI = confidence interval; *LL* = lower limit; *UL* = upper limit; VAS = Visual Analogue Scale

**Table 4:** Association between VRS itch score and clinical variables (*n* = 413)

VRS average itch within the past 24 h (0 = no itch, 1 = mild, 2 = moderate, 3 = severe, 4 = extremely severe)	Coef.	SE	95% CI		*p*
			*LL*	*UL*	
(Intercept)	-0.185	0.442	-1.051	0.681	0.676
**Age** (years)	0.005	0.005	-0.005	0.015	0.332
**Sex** Woman	0.053	0.141	-0.224	0.330	0.707
**Physician-diagnosed depression**	0.675	0.224	0.235	1.114	0.003
**Number of prescribed medications**	0.033	0.014	0.004	0.061	0.023

**Random effects**	Estimate	SE	95% CI		
Dialysis centers			*LL*	*UL*	
Constant Variance	0.072	0.048	0.020	0.264	
Residual Variance	1.787	0.126	1.556	2.052	
Likelihood Ratio (LR) χ²= 7.33, *p* = 0.0034					

**VRS worst itch** within the past 24 h (0 = no itch, 1 = mild, 2 = moderate, 3 = severe, 4 = extremely severe)	Coef.	*SE*	95% CI		*p*
			*LL*	*UL*	
(Intercept)	-0.193	0.559	-1.288	0.902	0.730
**Age** (years)	0.006	0.006	-0.006	0.019	0.321
**Sex** Woman	0.101	0.179	-0.249	0.451	0.573
**Physician-diagnosed depression**	0.787	0.284	0.231	1.342	0.006
**Number of prescribed medications**	0.038	0.018	0.002	0.074	0.036

**Random effects**	Estimate	SE	95% CI		
Dialysis centers			*LL*	*UL*	
Constant Variance	0.113	0.075	0.031	0.418	
Residual Variance	2.857	0.202	2.488	3.281	
Likelihood Ratio (LR) χ² = 7.2, *p* = 0.0037					

Analysis accounted for variations between dialysis centers by using a random intercept in the regression model. * Significance at *p* ≤ 0.05. Coef. = Coefficient; SE = Standard Error; CI = confidence interval; *LL* = lower limit; *UL* = upper limit; VRS = Verbal Rating Scale

The mixed-effects linear model did not show significant associations between the prevalence or severity of CKD-aP and age, sex, diabetes, use of HDF versus HD (-0.071, SE 0.05, 95% CI -0.170, 0.027, *p* = 0.157), eKt/V (-0.043, SE 0.070, 95% CI -0.180, 0.094, *p* = 0.54), volume of substitution, use of a tunneled catheter versus a fistula, frequency of dialysis sessions (n/week), dialysis session duration (in hours) and CRP (Supplementary Tables 1–5).

### CKD-aP and prescribed treatments

**Fig. 3:**
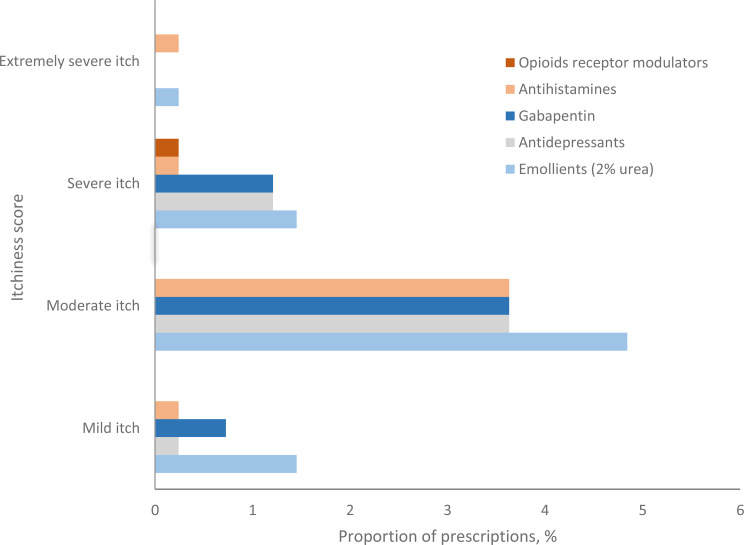
Prescription percentage of medications and emollients by VRS itch category among participants with CKD-aP (*n* = 101)

Topical emollients were the most prescribed CKD-aP treatment across all severity categories, while Opioid Receptor Modulators (Butorphanol, Naltrexone) were the least (Fig. 3). 25% of participants with CKD-aP did not receive anti-pruritic medications.

## Discussion

In this Swiss study, CKD-aP prevalence was 25%, with a significant variation between participating centers, despite similar patient characteristics and treatment approaches. This overall prevalence is lower than the rates reported in the literature, including data from DOPPS, which gathered information from neighboring countries [4].

The reason for the low CKD-aP prevalence in our study compared to surrounding countries remains unclear. Although HDF enhances the removal of middle-sized uremic toxins [22] implicated in CKD-aP via inflammatory and neuroimmune pathways [4], neither HDF nor the amount of volume substitution were associated with CKD-aP in our study. Therefore, the widespread use of HDF in Switzerland (73% in our study) does not explain the low prevalence of CKD-aP in our study. However, given the cross-sectional design, we cannot rule out indication bias; for example, HDF may have been prescribed in some cases for CKD-aP treatment. In the CONVINCE trial, participants were randomized to conventional HD or HDF. While HDF was associated with slower decline in patient-reported physical function and social activities compared to HD, CKD-aP was not assessed as endpoint [23]. Therefore, more randomized studies are needed to assess whether HDF lowers CKD-aP.

Reasons for the variability in CKD-aP prevalence across centers also remain unclear. Mean age, participants characteristics (e.g., sex, diabetes) and clinical variables were similar in centers with the highest (45%) and lowest (8%) prevalence. Of interest, the center with the lowest prevalence had the highest use of emollients, suggesting a potential beneficial effect. This difference may of course also be due to factors not investigated in our study such as diet and non-pharmacological approaches.

Regarding the severity of CKD-aP, in our study, 15% reported moderate and 5% severe itch, compared to 18% and 12% respectively in DOPPS [11]. Similarly, a USA study reported 15.3% moderate and 9.3% severe itch [12].

Participants with moderate itch most often used emollients, followed by gabapentin, antidepressants, and antihistamines, despite the reported limited efficacy of the latter [15]. Antidepressants, prescribed to 16% of participants, likely addressed both depression and CKD-aP. However, we were unable to determine the specific indication for these medications; they may have been prescribed for other comorbidities. Therefore, their use should not be interpreted as evidence of active CKD-aP. Kappa-opioid receptor agonists were unavailable in Switzerland during the study. A potential limitation is the classification of participants with VRS = 1 as having ‘no pruritus’ based solely on self-report, despite some receiving medications typically indicated for itch. This approach may underestimate the prevalence of CKD-aP when symptoms were well-controlled at assessment. However, our primary analysis relied on patient-reported severity, in line with the use of PROMs for symptom evaluation.

Gender and age were not associated with the prevalence or severity of CKD-aP in our study. This finding aligns with a study that found no gender differences [24], although another reported a higher prevalence among men [2]. Similarly, while we observed no association with age, one previous study linked increased age to moderate and severe CKD-aP [2], whereas another found a higher likelihood of CKD-aP in patients under 70 years [24].

Depression was the only clinical factor significantly associated with both CKD-aP prevalence and severity, consistent with DOPPS findings linking moderate to severe CKD-aP to physician-diagnosed depression [2]. In contrast, the GEHIS study, using Itch-related QoL and VAS scales over six weeks and Hospital Anxiety and Depression Scale (HADS) for depression, found no such association [25], likely due to differing assessment tools. Depression prevalence in our study (17%) was slightly higher than reported in DOPPS (13%). This higher prevalence, despite a lower CKD-aP prevalence, reflects the multifactorial nature of depression in dialysis patients, with CKD-aP as one of several contributing factors. Variations in population socioeconomic characteristics, comorbidities, healthcare access and context may further explain this discrepancy. The relationship between CKD-aP and depression is likely bidirectional. CKD-aP may trigger or worsen depression, and depression may aggravate CKD-aP [4, 26]. Since itching disrupts sleep and reduces overall QoL [27], the psychological burden of CKD-aP can lead to depression [4].

Based on previous literature, we expected that higher CRP, phosphorus, calcium, and potassium would be linked to higher CKD-aP risk [4, 11, 15, 16], and that decreased serum albumin, hemoglobin, and residual clearance would be associated with an increased CKD-aP risk. In our findings, only serum phosphorus levels were significantly associated with CKD-aP severity, not with its prevalence. The mean phosphorus level was lower than that reported by DOPPS [2, 11, 26], which may explain the weaker association in our study [11]. Although higher CRP levels were observed among participants with CKD-aP, statistical analysis revealed no significant association between CRP and either the prevalence or severity of CKD-aP. This lack of association in our data could be due to the high variability of CRP and the cross-sectional nature of our study, capturing CRP at a single time point, which may not reflect the chronic inflammatory burden relevant to CKD-aP pathogenesis. Moreover, high-sensitivity CRP, a more sensitive marker of chronic low-grade inflammation, was not available.

No significant associations were found with PTH or other biochemical parameters. These parameters were not more tightly controlled than in DOPPS (Table 1). Our study was smaller than DOPPS, and less powered to find weak associations between CKD-aP and biochemical or clinical variables. Therefore, our findings suggest that biochemical parameters including PTH do not play a major role in CKD-aP prevalence and severity.

BMI had a significant effect on CKD-aP prevalence, though the correlation coefficient was small. Previous research also linked higher BMI to increased CKD-aP risk [28]. Higher BMI may contribute to skin barrier dysfunction, dryness, inflammation and impaired thermoregulation.

Smoking was associated with increased CKD-aP severity based on the VAS–Worst Intensity score, but not other measures, aligning with previous studies [29]. Smoking is known to induce systemic inflammation and oxidative stress, which can exacerbate CKD-aP.

Unlike many previous studies, we examined the effect of polypharmacy on CKD-aP and itch severity. While polypharmacy was not associated with increased CKD-aP risk, it was associated with greater severity on VAS and VRS, though with small effect sizes. CKD patients often face a high medication burden, averaging 9 daily medications, ranging from 2 to 24 per day [30]. Colombijn et al. (2021) investigated polypharmacy impact on dialysis patients QoL, showing that a higher number of medications was associated with significantly lower SF-36 scores, poorer overall health perceptions and increased reported symptoms [31]. Managing multiple medications may increase stress, worsening CKD-aP via neurogenic inflammation.

To better capture the dose–response relationship between medication burden and symptom severity, we treated polypharmacy as a continuous variable (pills/day) in the statistical analysis. This approach considers more variability than threshold-based definitions of polypharmacy as ≥ 5 pills/day and reflects the substantial pill burden in our cohort (mean 15.3 pills/day, SD 4.9). As over 95% of participants met conventional polypharmacy criteria for high pill burden, a binary classification would have limited interpretability and statistical power.

The discrepancy in prevalence and associated factors between the DOPPS findings and our results may also be attributed to the shorter assessment period and the different nature of the questions. While Rayner et al. asked participants, “To what extent were you bothered by itchy skin during the past 4 weeks?” [4], our study focused on symptoms experienced within the past 24 h. Our use of a 24-hour recall window was chosen to improve symptom recall accuracy and minimize bias. While this may lead to lower prevalence estimates compared to studies using longer recall periods such as DOPPS (4 weeks), it offers a more immediate and reliable assessment of current symptom burden. We utilized face-to-face interviews by a research nurse, which may have reduced bias compared to the general questionnaire used by DOPPS. We only considered itchiness specifically attributed to CKD-aP in the data collection. The DOPPS study might have included some false positives due to the broader range of participants, including those with skin conditions that could report itchiness unrelated to CKD-aP. However, the number of patients with an alternative skin condition was low in our study.

In summary, our findings show that CKD-aP prevalence, while lower than in neighboring countries, still persistently affects one in four Swiss HD patients despite the use of modern dialysis techniques, supporting holistic care targeting mental health, smoking, BMI, and polypharmacy. While phosphorus may not trigger CKD-aP, it worsens severity, supporting phosphate reduction to KDIGO-goals [17].

This study provides valuable insights into CKD-aP prevalence and risk factors under high-standard care, but its cross-sectional design precludes causal inference. Accordingly, associations observed, particularly those involving depression and phosphorus levels, should not be interpreted as causal. The lower CKD-aP prevalence in Switzerland remains hypothetical compared to neighboring countries and findings may not be generalized to healthcare settings with different standards.

Longitudinal studies are needed to evaluate long-term effects of multidisciplinary interventions and opioid receptor modulators on CKD-aP, sleep and QoL.

## Conclusion

Despite high dialysis standards in Switzerland and the use of HDF, 25% of HD patients still experience CKD-aP. While associations with depression and phosphorus were identified, these are not indicative of causality given the cross-sectional design. Depression is significantly associated with CKD-aP and itch severity, while smoking, high phosphorus levels, and polypharmacy worsen itch severity. The link between CKD-aP and depression highlights the need for a holistic approach. CKD-aP management can be improved through depression screening, phosphate level control, dialysis optimization, dietary restrictions, smoking cessation, deprescribing unnecessary medications and considering new management options such as Difelikefalin.

## Supplementary Information

Below is the link to the electronic supplementary material.

Supplementary Material 1

## Data Availability

Data will be shared on reasonable request to the corresponding author.

## Abbreviations

CKD-aP: Chronic Kidney Disease-Associated Pruritus
DOPPS: Dialysis Outcomes and Practice Patterns Study
HDF: Hemodiafiltration
HD: Hemodialysis
NRS: Numeric Rating Scale
QoL: Quality of Life
VRS: Verbal Rating Scale
VAS: Visual Analogue Scale
