# Supplementary material for: Prevalence, severity, and determinants of CKD-associated pruritus in a Swiss hemodialysis population with widespread use of hemodiafiltration: a cross-sectional study
Source: BMC Nephrol. 2025 Nov 17;26:640. doi: 10.1186/s12882-025-04570-w (PMC12625539; doi:10.1186/s12882-025-04570-w)
Supplement: Supplementary file 1 — Supplementary Material 1 [file 12882_2025_4570_MOESM1_ESM.docx]

**Supplementary tables of random effect models (REM)**

SupplementaryTable 1. CKD-aP presence and independent variables REM

| Sociodemographic / clinical variable | n | Coef. | *SE* | 95% CI | | ***p*** | Random Effect Var. |  | Residual Var. |  | LR Test  χ² |
| --- | --- | --- | --- | --- | --- | --- | --- | --- | --- | --- | --- |
|  |  |  |  | *LL* | *UL* |  |  | *SD* |  | SD |  |
| Age (years) | 413 | 0.002 | 0.002 | -0.001 | 0.005 | 0.235 | 0.005 | 0.004 | 0.179 | 0.013 | 4.27 |
| Sex | 413 | 0.006 | 0.044 | -0.081 | 0.093 | 0.895 | 0.005 | 0.004 | 0.180 | 0.013 | 3.8 |
| Marital Status (reference: Single) | 413 |  |  |  |  |  |  |  |  |  |  |
| Married |  | -0.053 | 0.060 | -0.170 | 0.064 | 0.377 | 0.005 | 0.004 | 0.177 | 0.013 | 4.2 |
| In a relationship |  | -0.313 | 0.218 | -0.740 | 0.114 | 0.151 |  |  |  |  |  |
| Divorced |  | -0.134 | 0.076 | -0.284 | 0.015 | 0.079 |  |  |  |  |  |
| Widowed |  | -0.032 | 0.073 | -0.176 | 0.111 | 0.660 |  |  |  |  |  |
| Separated |  | -0.160 | 0.168 | -0.489 | 0.170 | 0.343 |  |  |  |  |  |
| Living alone | 413 | 0.011 | 0.045 | -0.077 | 0.100 | 0.801 | 0.005 | 0.004 | 0.180 | 0.013 | 3.76 |
| BMI (kg/m2) | 413 | 0.008 | 0.004 | 0.000 | 0.015 | 0.047***** | 0.005 | 0.004 | 0.178 | 0.013 | 4.35 |
| Smoker | 413 | 0.086 | 0.055 | -0.021 | 0.193 | 0.113 | 0.004 | 0.003 | 0.179 | 0.013 | 2.62 |
| No. of prescribed medications | 413 | 0.008 | 0.005 | -0.001 | 0.017 | 0.088 | 0.006 | 0.004 | 0.178 | 0.013 | 4.97 |
| No. of years on dialysis (years) | 412 | 0.002 | 0.004 | -0.006 | 0.011 | 0.562 | 0.004 | 0.004 | 0.180 | 0.013 | 3.59 |
| Diabetes | 413 | 0.013 | 0.042 | -0.070 | 0.096 | 0.757 | 0.005 | 0.004 | 0.180 | 0.013 | 3.83 |
| Peripheral Vascular Disease | 413 | 0.032 | 0.042 | -0.051 | 0.115 | 0.455 | 0.005 | 0.004 | 0.179 | 0.013 | 3.79 |
| Severe Covid-19 complications | 413 | 0.149 | 0.105 | -0.058 | 0.356 | 0.158 | 0.005 | 0.023 | 0.178 | 0.205 | 4.62 |
| Physician-diagnosed depression | 413 | 0.179 | 0.070 | 0.042 | 0.316 | 0.010***** | 0.004 | 0.003 | 0.177 | 0.012 | 3.07 |
| Dialysis Treatment (reference: HDF) | 413 |  |  |  |  |  |  |  |  |  |  |
| HD |  | -0.071 | 0.050 | -0.170 | 0.027 | 0.157 | 0.006 | 0.004 | 0.178 | 0.013 | 4.96 |
| Volume substitution (L) | 248 | -0.006 | 0.004 | -0.013 | 0.002 | 0.157 | 0.009 | 0.008 | 0.175 | 0.016 | 3.76 |
| Vascular access (reference: Fistula) | 413 |  |  |  |  |  |  |  |  |  |  |
| Permcath |  | 0.008 | 0.044 | -0.078 | 0.095 | 0.849 | 0.005 | 0.004 | 0.180 | 0.013 | 3.82 |
| Frequency of dialysis (n/week) | 413 | -0.054 | 0.066 | -0.184 | 0.075 | 0.412 | 0.005 | 0.004 | 0.179 | 0.013 | 4.28 |
| Dialysis session duration (hours) | 413 | 0.008 | 0.060 | -0.110 | 0.126 | 0.894 | 0.005 | 0.004 | 0.180 | 0.013 | 3.71 |
| eKt/V | 371 | -0.043 | 0.070 | -0.180 | 0.094 | 0.540 | 0.003 | 0.003 | 0.188 | 0.014 | 2.01 |
| Serum albumin (g/L) | 408 | -0.002 | 0.005 | -0.011 | 0.007 | 0.688 | 0.005 | 0.004 | 0.180 | 0.013 | 3.99 |
| Urea (mmol/L) | 413 | -0.002 | 0.002 | -0.005 | 0.001 | 0.237 | 0.004 | 0.004 | 0.179 | 0.013 | 3.54 |
| Residual Clearance (%) | 32 | -0.002 | 0.013 | -0.028 | 0.023 | 0.870 | 0.000 | 0.000 | 0.234 | 0.059 | 0.00 |
| CRP (mg/L) | 320 | 0.000 | 0.001 | -0.001 | 0.002 | 0.584 | 0.004 | 0.004 | 0.184 | 0.015 | 1.85 |
| Hemoglobin (g/L) | 413 | -0.002 | 0.002 | -0.006 | 0.001 | 0.182 | 0.005 | 0.004 | 0.179 | 0.013 | 3.82 |
| Phosphorus (mmol/L) | 411 | 0.056 | 0.051 | -0.045 | 0.156 | 0.277 | 0.005 | 0.004 | 0.179 | 0.013 | 4.53 |
| Calcium (mmol/L) | 412 | 0.171 | 0.133 | -0.090 | 0.431 | 0.199 | 0.004 | 0.004 | 0.180 | 0.013 | 3.21 |
| Potassium (mmol/L) | 413 | -0.002 | 0.032 | -0.065 | 0.060 | 0.939 | 0.005 | 0.004 | 0.180 | 0.013 | 3.61 |
| y-GT (U/L) | 370 | 0.001 | 0.000 | 0.000 | 0.001 | 0.097 | 0.006 | 0.004 | 0.170 | 0.013 | 3.93 |
| Total Bilirubin µmol/L | 139 | -0.002 | 0.004 | -0.010 | 0.007 | 0.676 | 0.019 | 0.013 | 0.143 | 0.018 | 11.44 |
| Ferritin (µg/L) | 403 | 0.000 | 0.000 | 0.000 | 0.000 | 0.165 | 0.005 | 0.004 | 0.181 | 0.013 | 3.54 |
| PTH (pmol/L) | 398 | 0.000 | 0.001 | -0.001 | 0.002 | 0.626 | 0.004 | 0.004 | 0.183 | 0.013 | 3.28 |
| Filter composition (reference: AN69) | 413 |  |  |  |  |  |  |  |  |  |  |
| Modified cellulose |  | -0.139 | 0.435 | -0.991 | 0.713 | 0.749 | 0.005 | 0.004 | 0.178 | 0.013 | 4.57 |
| MCO |  | 0.242 | 0.213 | -0.175 | 0.659 | 0.256 |  |  |  |  |  |
| Polyethersulfone |  | 0.087 | 0.121 | -0.151 | 0.324 | 0.475 |  |  |  |  |  |
| Polysulfone |  | 0.100 | 0.096 | -0.087 | 0.288 | 0.294 |  |  |  |  |  |
| Triacetate |  | 0.187 | 0.143 | -0.093 | 0.468 | 0.191 |  |  |  |  |  |

* Significance at p ≤ 0.05

Supplementary Table 2. CKD-aP VAS - Average itch score within the past 24h and independent variables REM

| Sociodemographic / clinical variable | n | Coef. | *SE* | 95% CI | | ***p*** | Random Effect Var. |  | Residual Var. |  | LR Test  χ² |
| --- | --- | --- | --- | --- | --- | --- | --- | --- | --- | --- | --- |
|  |  |  |  | *LL* | *UL* |  |  | *SD* |  | SD |  |
| Age (years) | 413 | 0.004 | 0.008 | -0.012 | 0.020 | 0.647 | 0.236 | 0.143 | 4.844 | 0.342 | 10.24 |
| Sex | 413 | 0.259 | 0.230 | -0.192 | 0.709 | 0.261 | 0.233 | 0.141 | 4.832 | 0.341 | 10.25 |
| Marital Status (reference: Single) | 413 |  |  |  |  |  |  |  |  |  |  |
| Married |  | -0.254 | 0.311 | -0.864 | 0.356 | 0.414 | 0.239 | 0.143 | 4.792 | 0.338 | 10.66 |
| In a relationship |  | -1.465 | 1.135 | -3.689 | 0.760 | 0.197 |  |  |  |  |  |
| Divorced |  | -0.561 | 0.398 | -1.342 | 0.220 | 0.159 |  |  |  |  |  |
| Widowed |  | -0.061 | 0.382 | -0.809 | 0.687 | 0.873 |  |  |  |  |  |
| Separated |  | -1.005 | 0.876 | -2.722 | 0.712 | 0.251 |  |  |  |  |  |
| Living alone | 413 | 0.069 | 0.236 | -0.393 | 0.531 | 0.769 | 0.230 | 0.140 | 4.848 | 0.342 | 9.94 |
| BMI (kg/m^2^) | 413 | 0.017 | 0.020 | -0.022 | 0.056 | 0.397 | 0.237 | 0.143 | 4.837 | 0.341 | 10.38 |
| Smoker | 413 | 0.525 | 0.285 | -0.034 | 1.084 | 0.066 | 0.200 | 0.129 | 4.821 | 0.340 | 8.07 |
| No. of prescribed medications | 413 | 0.067 | 0.024 | 0.021 | 0.114 | 0.004***** | 0.293 | 0.167 | 4.732 | 0.334 | 13.47 |
| No. of years on dialysis (years) | 412 | 0.025 | 0.022 | -0.017 | 0.068 | 0.243 | 0.224 | 0.138 | 0.067 | 0.747 | 9.66 |
| Diabetes | 413 | 0.101 | 0.221 | -0.334 | 0.535 | 0.650 | 0.232 | 0.141 | 4.845 | 0.342 | 10.11 |
| Peripheral Vascular Disease | 413 | 0.093 | 0.221 | -0.339 | 0.525 | 0.673 | 0.231 | 0.140 | 4.846 | 0.342 | 10.03 |
| Severe Covid-19 complications | 413 | 0.581 | 0.550 | -0.496 | 1.659 | 0.290 | 0.245 | 0.146 | 4.829 | 0.341 | 10.81 |
| Physician-diagnosed depression | 413 | 1.279 | 0.361 | 0.571 | 1.987 | 0.000***** | 0.200 | 0.127 | 4.716 | 0.333 | 8.64 |
| Dialysis Treatment (reference: HDF) | 413 |  |  |  |  |  |  |  |  |  |  |
| HD |  | -0.433 | 0.266 | -0.955 | 0.089 | 0.104 | 0.270 | 0.158 | 4.803 | 0.339 | 11.99 |
| Volume substitution (L) | 248 | -0.034 | 0.020 | -0.074 | 0.005 | 0.091 | 0.467 | 0.303 | 4.641 | 0.426 | 10.07 |
| Vascular access (reference: Fistula) | 413 |  |  |  |  |  |  |  |  |  |  |
| Permcath |  | -0.028 | 0.229 | -0.478 | 0.422 | 0.902 | 0.230 | 0.140 | 4.848 | 0.342 | 10.00 |
| Frequency of dialysis (n/week) | 413 | -0.338 | 0.345 | -1.015 | 0.338 | 0.327 | 0.249 | 0.148 | 4.830 | 0.341 | 10.86 |
| Dialysis session duration (hours) | 413 | -0.109 | 0.318 | -0.733 | 0.515 | 0.732 | 0.236 | 0.143 | 4.845 | 0.342 | 10.15 |
| eKt/V | 371 | -0.088 | 0.374 | -0.820 | 0.644 | 0.814 | 0.195 | 0.137 | 5.216 | 0.388 | 6.35 |
| Serum albumin (g/L) | 408 | 0.006 | 0.025 | -0.043 | 0.055 | 0.807 | 0.245 | 0.144 | 4.682 | 0.332 | 11.53 |
| Urea (mmol/L) | 413 | -0.011 | 0.009 | -0.028 | 0.006 | 0.202 | 0.219 | 0.135 | 4.834 | 0.341 | 9.47 |
| Residual Clearance (%) | 32 | -0.015 | 0.079 | -0.171 | 0.141 | 0.848 | 0.000 | 0.000 | 8.740 | 2.185 | 0.00 |
| CRP (mg/L) | 320 | 0.000 | 0.005 | -0.009 | 0.010 | 0.936 | 0.219 | 0.150 | 5.020 | 0.403 | 6.41 |
| Hemoglobin (g/L) | 413 | -0.009 | 0.009 | -0.026 | 0.008 | 0.301 | 0.230 | 0.140 | 4.836 | 0.341 | 10.00 |
| Phosphorus (mmol/L) | 411 | 0.423 | 0.266 | -0.100 | 0.945 | 0.113 | 0.256 | 0.150 | 4.826 | 0.341 | 11.6 |
| Calcium (mmol/L) | 412 | 0.901 | 0.697 | -0.464 | 2.266 | 0.196 | 0.221 | 0.137 | 4.842 | 0.342 | 9.34 |
| Potassium (mmol/L) | 413 | 0.064 | 0.166 | -0.260 | 0.389 | 0.697 | 0.237 | 0.143 | 4.844 | 0.342 | 10.17 |
| y-GT (U/L) | 370 | 0.002 | 0.002 | -0.002 | 0.005 | 0.320 | 0.274 | 0.165 | 4.505 | 0.337 | 9.99 |
| Total Bilirubin µmol/L | 139 | -0.013 | 0.024 | -0.061 | 0.034 | 0.577 | 0.723 | 0.458 | 4.264 | 0.523 | 16.94 |
| Ferritin (µg/L) | 403 | 0.000 | 0.000 | 0.000 | 0.001 | 0.168 | 0.233 | 0.142 | 4.829 | 0.345 | 9.89 |
| PTH (pmol/L) | 398 | 0.005 | 0.005 | -0.005 | 0.014 | 0.339 | 0.226 | 0.141 | 4.965 | 0.357 | 9.1 |
| Filter composition (reference: AN69) | 413 |  |  |  |  |  |  |  |  |  |  |
| Modified cellulose |  | -0.292 | 2.261 | -4.724 | 4.140 | 0.897 | 0.267 | 0.157 | 4.799 | 0.339 | 11.75 |
| MCO |  | 0.649 | 1.114 | -1.533 | 2.831 | 0.560 |  |  |  |  |  |
| Polyethersulfone |  | 0.587 | 0.642 | -0.672 | 1.846 | 0.361 |  |  |  |  |  |
| Polysulfone |  | 0.762 | 0.501 | -0.220 | 1.744 | 0.128 |  |  |  |  |  |
| Triacetate |  | 1.071 | 0.750 | -0.398 | 2.541 | 0.153 |  |  |  |  |  |

* Significance at p ≤ 0.05

Supplementary Table 3. CKD-aP VAS - Worst itch score within the past 24h and independent variables REM

| Sociodemographic / clinical variable | n | Coef. | *SE* | 95% CI | | *p* | Random Effect Var. |  | Residual Var. |  | LR Test  χ² |
| --- | --- | --- | --- | --- | --- | --- | --- | --- | --- | --- | --- |
|  |  |  |  | *LL* | *UL* |  |  | *SD* |  | SD |  |
| Age (years) | 413 | -0.003 | 0.011 | -0.024 | 0.019 | 0.822 | 0.287 | 0.209 | 8.932 | 0.630 | 5.28 |
| Sex | 413 | 0.298 | 0.312 | -0.313 | 0.909 | 0.340 | 0.294 | 0.210 | 8.910 | 0.629 | 5.57 |
| Marital Status (reference: Single) | 413 |  |  |  |  |  |  |  |  |  |  |
| Married |  | -0.595 | 0.422 | -1.421 | 0.231 | 0.158 | 0.298 | 0.211 | 8.819 | 0.622 | 5.79 |
| In a relationship |  | -2.292 | 1.538 | -5.306 | 0.723 | 0.136 |  |  |  |  |  |
| Divorced |  | -0.961 | 0.540 | -2.019 | 0.097 | 0.075 |  |  |  |  |  |
| Widowed |  | -0.430 | 0.517 | -1.444 | 0.584 | 0.406 |  |  |  |  |  |
| Separated |  | -1.005 | 1.187 | -3.332 | 1.322 | 0.397 |  |  |  |  |  |
| Living alone | 413 | 0.189 | 0.319 | -0.437 | 0.814 | 0.555 | 0.287 | 0.208 | 8.926 | 0.630 | 5.33 |
| BMI (kg/m^2^) | 413 | 0.041 | 0.027 | -0.012 | 0.093 | 0.130 | 0.311 | 0.218 | 8.872 | 0.626 | 6.01 |
| Smoker | 413 | 0.922 | 0.384 | 0.169 | 1.675 | 0.016***** | 0.225 | 0.183 | 8.843 | 0.624 | 3.65 |
| No. of prescribed medications | 413 | 0.086 | 0.032 | 0.023 | 0.148 | 0.007***** | 0.400 | 0.257 | 8.732 | 0.617 | 8.29 |
| No. of years on dialysis (years) | 412 | 0.027 | 0.029 | -0.030 | 0.085 | 0.354 | 0.281 | 0.205 | 8.935 | 0.631 | 5.16 |
| Diabetes | 413 | 0.006 | 0.300 | -0.582 | 0.594 | 0.983 | 0.291 | 0.210 | 8.932 | 0.630 | 5.43 |
| Peripheral Vascular Disease | 413 | 0.165 | 0.299 | -0.420 | 0.751 | 0.580 | 0.290 | 0.209 | 8.925 | 0.630 | 5.45 |
| Severe Covid-19 complications | 413 | 1.212 | 0.744 | -0.246 | 2.669 | 0.103 | 0.325 | 0.223 | 8.858 | 0.625 | 6.47 |
| Physician-diagnosed depression | 413 | 1.701 | 0.490 | 0.741 | 2.661 | 0.001***** | 0.246 | 0.188 | 8.698 | 0.613 | 4.51 |
| Dialysis Treatment (reference: HDF) | 413 |  |  |  |  |  |  |  |  |  |  |
| HD |  | -0.522 | 0.357 | -1.222 | 0.177 | 0.143 | 0.342 | 0.232 | 8.862 | 0.626 | 6.73 |
| Volume substitution (L) | 248 | -0.054 | 0.028 | -0.109 | 0.001 | 0.055 | 0.606 | 0.458 | 9.053 | 0.831 | 5.72 |
| Vascular access (reference: Fistula) | 413 |  |  |  |  |  |  |  |  |  |  |
| Permcath |  | 0.054 | 0.311 | -0.554 | 0.663 | 0.861 | 0.292 | 0.211 | 8.930 | 0.630 | 5.46 |
| Frequency of dialysis (n/week) | 413 | -0.397 | 0.467 | -1.312 | 0.518 | 0.395 | 0.317 | 0.221 | 8.904 | 0.628 | 6.02 |
| Dialysis session duration (hours) | 413 | -0.012 | 0.427 | -0.849 | 0.825 | 0.978 | 0.291 | 0.211 | 8.931 | 0.630 | 5.40 |
| eKt/V | 371 | -0.202 | 0.501 | -1.183 | 0.779 | 0.686 | 0.230 | 0.203 | 9.535 | 0.710 | 3.06 |
| Serum albumin (g/L) | 408 | -0.004 | 0.034 | -0.070 | 0.062 | 0.908 | 0.308 | 0.215 | 8.824 | 0.626 | 6.07 |
| Urea (mmol/L) | 413 | -0.013 | 0.012 | -0.036 | 0.011 | 0.284 | 0.275 | 0.202 | 8.915 | 0.629 | 5.06 |
| Residual Clearance (%) | 32 | -0.004 | 0.092 | -0.185 | 0.176 | 0.962 | 0.000 | 0.000 | 11.748 | 2.937 | 0.00 |
| CRP (mg/L) | 320 | -0.000 | 0.006 | -0.013 | 0.013 | 0.988 | 0.241 | 0.219 | 9.224 | 0.741 | 2.62 |
| Hemoglobin (g/L) | 413 | -0.008 | 0.012 | -0.032 | 0.015 | 0.478 | 0.290 | 0.209 | 8.921 | 0.629 | 5.43 |
| Phosphorus (mmol/L) | 411 | 0.818 | 0.359 | 0.114 | 1.522 | 0.023***** | 0.352 | 0.230 | 8.823 | 0.624 | 7.48 |
| Calcium (mmol/L) | 412 | 0.772 | 0.941 | -1.072 | 2.617 | 0.412 | 0.275 | 0.204 | 8.942 | 0.632 | 4.93 |
| Potassium (mmol/L) | 413 | 0.109 | 0.224 | -0.330 | 0.549 | 0.626 | 0.304 | 0.217 | 8.920 | 0.629 | 5.67 |
| y-GT (U/L) | 370 | 0.003 | 0.002 | -0.002 | 0.007 | 0.226 | 0.302 | 0.227 | 8.307 | 0.621 | 4.62 |
| Total Bilirubin µmol/L | 139 | -0.015 | 0.031 | -0.076 | 0.045 | 0.620 | 0.944 | 0.648 | 7.017 | 0.860 | 11.87 |
| Ferritin (µg/L) | 403 | 0.000 | 0.000 | 0.000 | 0.001 | 0.231 | 0.297 | 0.214 | 8.920 | 0.637 | 5.40 |
| PTH (pmol/L) | 398 | 0.005 | 0.006 | -0.008 | 0.017 | 0.477 | 0.287 | 0.213 | 9.131 | 0.656 | 4.94 |
| Filter composition (reference: AN69) | 413 |  |  |  |  |  |  |  |  |  |  |
| Modified cellulose |  | -0.961 | 3.071 | -6.980 | 5.058 | 0.754 | 0.331 | 0.230 | 8.874 | 0.627 | 6.24 |
| MCO |  | 1.215 | 1.507 | -1.739 | 4.168 | 0.420 |  |  |  |  |  |
| Polyethersulfone |  | 0.302 | 0.862 | -1.388 | 1.992 | 0.726 |  |  |  |  |  |
| Polysulfone |  | 0.561 | 0.677 | -0.767 | 1.889 | 0.408 |  |  |  |  |  |
| Triacetate |  | 1.008 | 1.013 | -0.978 | 2.994 | 0.320 |  |  |  |  |  |

* Significance at p ≤ 0.05

Supplementary Table 4. CKD-aP VRS - Average itch score within the past 24h and independent variables REM

| Sociodemographic / clinical variable | n | Coef. | *SE* | 95% CI | | *p* | Random Effect Var. |  | Residual Var. |  | LR Test  χ² |
| --- | --- | --- | --- | --- | --- | --- | --- | --- | --- | --- | --- |
|  |  |  |  | *LL* | *UL* |  |  | *SD* |  | SD |  |
| Age (years) | 413 | 0.003 | 0.005 | -0.007 | 0.013 | 0.594 | 0.065 | 0.045 | 1.859 | 0.131 | 6.17 |
| Sex | 413 | 0.096 | 0.142 | -0.183 | 0.375 | 0.502 | 0.064 | 0.045 | 1.858 | 0.131 | 6.05 |
| Marital Status (reference: Single) | 413 |  |  |  |  |  |  |  |  |  |  |
| Married |  | -0.199 | 0.192 | -0.577 | 0.178 | 0.300 | 0.066 | 0.045 | 1.836 | 0.130 | 6.47 |
| In a relationship |  | -0.999 | 0.702 | -2.375 | 0.376 | 0.155 |  |  |  |  |  |
| Divorced |  | -0.424 | 0.246 | -0.906 | 0.059 | 0.085 |  |  |  |  |  |
| Widowed |  | -0.098 | 0.236 | -0.561 | 0.364 | 0.677 |  |  |  |  |  |
| Separated |  | -0.543 | 0.542 | -1.605 | 0.519 | 0.316 |  |  |  |  |  |
| Living alone | 413 | 0.055 | 0.146 | -0.230 | 0.341 | 0.704 | 0.063 | 0.044 | 1.860 | 0.131 | 5.87 |
| BMI (kg/m^2^) | 413 | 0.018 | 0.012 | -0.006 | 0.042 | 0.132 | 0.067 | 0.046 | 1.849 | 0.130 | 6.46 |
| Smoker | 413 | 0.283 | 0.176 | -0.062 | 0.628 | 0.108 | 0.054 | 0.041 | 1.854 | 0.131 | 4.53 |
| No. of prescribed medications | 413 | 0.036 | 0.015 | 0.007 | 0.064 | 0.014***** | 0.080 | 0.051 | 1.827 | 0.129 | 8.14 |
| No. of years on dialysis (years) | 412 | 0.01 | 0.013 | -0.016 | 0.037 | 0.434 | 0.061 | 0.044 | 1.863 | 0.132 | 5.67 |
| Diabetes | 413 | 0.068 | 0.137 | -0.200 | 0.337 | 0.618 | 0.064 | 0.045 | 1.859 | 0.131 | 6.03 |
| Peripheral Vascular Disease | 413 | 0.104 | 0.136 | -0.164 | 0.371 | 0.447 | 0.063 | 0.044 | 1.858 | 0.131 | 5.97 |
| Severe Covid-19 complications | 413 | 0.495 | 0.340 | -0.171 | 1.161 | 0.145 | 0.070 | 0.047 | 1.848 | 0.130 | 6.92 |
| Physician-diagnosed depression | 413 | 0.706 | 0.224 | 0.267 | 1.146 | 0.002***** | 0.054 | 0.040 | 1.821 | 0.128 | 4.96 |
| Dialysis Treatment (reference: HDF) | 413 |  |  |  |  |  |  |  |  |  |  |
| HD |  | -0.216 | 0.163 | -0.535 | 0.104 | 0.186 | 0.073 | 0.049 | 1.848 | 0.130 | 7.14 |
| Volume substitution (L) | 248 | -0.021 | 0.013 | -0.046 | 0.003 | 0.092 | 0.133 | 0.097 | 1.813 | 0.166 | 6.39 |
| Vascular access (reference: Fistula) | 413 |  |  |  |  |  |  |  |  |  |  |
| Permcath |  | 0.037 | 0.142 | -0.241 | 0.315 | 0.794 | 0.064 | 0.045 | 1.860 | 0.131 | 6.00 |
| Frequency of dialysis (n/week) | 413 | -0.194 | 0.213 | -0.612 | 0.223 | 0.362 | 0.069 | 0.047 | 1.854 | 0.131 | 6.61 |
| Dialysis session duration (hours) | 413 | 0.014 | 0.195 | -0.368 | 0.397 | 0.941 | 0.063 | 0.044 | 1.861 | 0.131 | 5.87 |
| eKt/V | 371 | -0.166 | 0.228 | -0.613 | 0.281 | 0.467 | 0.050 | 0.043 | 1.973 | 0.147 | 3.42 |
| Serum albumin (g/L) | 408 | -0.005 | 0.015 | -0.035 | 0.025 | 0.743 | 0.068 | 0.046 | 1.828 | 0.130 | 6.85 |
| Urea (mmol/L) | 413 | -0.007 | 0.005 | -0.018 | 0.004 | 0.195 | 0.059 | 0.043 | 1.855 | 0.131 | 5.52 |
| Residual Clearance (%) | 32 | 0.008 | 0.045 | -0.079 | 0.095 | 0.856 | 0.000 | 0.000 | 2.747 | 0.687 | 0.00 |
| CRP (mg/L) | 320 | 0.001 | 0.003 | -0.004 | 0.007 | 0.664 | 0.061 | 0.049 | 1.888 | 0.152 | 3.83 |
| Hemoglobin (g/L) | 413 | -0.006 | 0.005 | -0.017 | 0.004 | 0.232 | 0.064 | 0.044 | 1.854 | 0.131 | 6.00 |
| Phosphorus (mmol/L) | 411 | 0.258 | 0.165 | -0.064 | 0.581 | 0.117 | 0.073 | 0.048 | 1.851 | 0.131 | 7.33 |
| Calcium (mmol/L) | 412 | 0.465 | 0.430 | -0.377 | 1.306 | 0.279 | 0.060 | 0.043 | 1.861 | 0.131 | 5.40 |
| Potassium (mmol/L) | 413 | 0.013 | 0.102 | -0.188 | 0.214 | 0.899 | 0.064 | 0.045 | 1.860 | 0.131 | 5.88 |
| y-GT (U/L) | 370 | 0.001 | 0.001 | -0.001 | 0.003 | 0.220 | 0.080 | 0.054 | 1.757 | 0.131 | 6.55 |
| Total Bilirubin µmol/L | 139 | -0.008 | 0.015 | -0.037 | 0.021 | 0.593 | 0.240 | 0.161 | 1.639 | 0.201 | 13.3 |
| Ferritin (µg/L) | 403 | 0.000 | 0.000 | 0.000 | 0.001 | 0.185 | 0.063 | 0.045 | 1.860 | 0.133 | 5.69 |
| PTH (pmol/L) | 398 | 0.003 | 0.003 | -0.003 | 0.008 | 0.383 | 0.062 | 0.045 | 1.898 | 0.136 | 5.31 |
| Filter composition (reference: AN69) | 413 |  |  |  |  |  |  |  |  |  |  |
| Modified cellulose |  | -0.393 | 1.401 | -3.139 | 2.354 | 0.779 | 0.071 | 0.048 | 1.847 | 0.130 | 6.76 |
| MCO |  | 0.696 | 0.688 | -0.653 | 2.044 | 0.312 |  |  |  |  |  |
| Polyethersulfone |  | 0.261 | 0.394 | -0.511 | 1.033 | 0.507 |  |  |  |  |  |
| Polysulfone |  | 0.325 | 0.309 | -0.281 | 0.931 | 0.293 |  |  |  |  |  |
| Triacetate |  | 0.541 | 0.463 | -0.366 | 1.447 | 0.243 |  |  |  |  |  |

* Significance at p ≤ 0.05

Supplementary Table 5. CKD-aP VRS– Worst itch score within the past 24h and independent variables REM

| Sociodemographic / clinical variable | n | Coef. | *SE* | 95% CI | | *p* | Random Effect Var. |  | Residual Var. |  | LR Test  χ² |
| --- | --- | --- | --- | --- | --- | --- | --- | --- | --- | --- | --- |
|  |  |  |  | *LL* | *UL* |  |  | *SD* |  | SD |  |
| Age (years) | 413 | 0.004 | 0.006 | -0.009 | 0.016 | 0.573 | 0.101 | 0.071 | 2.959 | 0.209 | 5.91 |
| Sex | 413 | 0.148 | 0.180 | -0.204 | 0.501 | 0.409 | 0.099 | 0.070 | 2.957 | 0.209 | 5.80 |
| Marital Status (reference: Single) | 413 |  |  |  |  |  |  |  |  |  |  |
| Married |  | -0.255 | 0.243 | -0.731 | 0.221 | 0.294 | 0.101 | 0.070 | 2.925 | 0.206 | 6.12 |
| In a relationship |  | -1.275 | 0.886 | -3.011 | 0.461 | 0.150 |  |  |  |  |  |
| Divorced |  | -0.525 | 0.311 | -1.135 | 0.084 | 0.091 |  |  |  |  |  |
| Widowed |  | -0.118 | 0.298 | -0.702 | 0.466 | 0.691 |  |  |  |  |  |
| Separated |  | -0.648 | 0.684 | -1.988 | 0.692 | 0.343 |  |  |  |  |  |
| Living alone | 413 | 0.092 | 0.184 | -0.269 | 0.452 | 0.619 | 0.097 | 0.069 | 2.962 | 0.209 | 5.61 |
| BMI (kg/m^2^) | 413 | 0.026 | 0.015 | -0.005 | 0.056 | 0.095 | 0.103 | 0.072 | 2.940 | 0.207 | 6.20 |
| Smoker | 413 | 0.407 | 0.222 | -0.028 | 0.842 | 0.067 | 0.081 | 0.063 | 2.948 | 0.208 | 4.22 |
| No. of prescribed medications | 413 | 0.042 | 0.018 | 0.006 | 0.078 | 0.023***** | 0.123 | 0.081 | 2.915 | 0.206 | 7.77 |
| No. of years on dialysis (years) | 412 | 0.014 | 0.017 | -0.020 | 0.047 | 0.422 | 0.094 | 0.068 | 2.966 | 0.209 | 5.38 |
| Diabetes | 413 | 0.014 | 0.173 | -0.325 | 0.353 | 0.935 | 0.098 | 0.070 | 2.963 | 0.209 | 5.70 |
| Peripheral Vascular Disease | 413 | 0.108 | 0.172 | -0.230 | 0.445 | 0.531 | 0.098 | 0.069 | 2.960 | 0.209 | 5.71 |
| Severe Covid-19 complications | 413 | 0.684 | 0.428 | -0.155 | 1.524 | 0.110 | 0.110 | 0.074 | 2.939 | 0.207 | 6.77 |
| Physician-diagnosed depression | 413 | 0.826 | 0.283 | 0.271 | 1.382 | 0.004***** | 0.086 | 0.064 | 2.908 | 0.205 | 4.92 |
| Dialysis Treatment (reference: HDF) | 413 |  |  |  |  |  |  |  |  |  |  |
| HD |  | -0.337 | 0.206 | -0.740 | 0.066 | 0.101 | 0.118 | 0.079 | 2.935 | 0.207 | 7.26 |
| Volume substitution (L) | 248 | -0.029 | 0.016 | -0.061 | 0.003 | 0.074 | 0.217 | 0.158 | 3.016 | 0.277 | 6.55 |
| Vascular access (reference: Fistula) | 413 |  |  |  |  |  |  |  |  |  |  |
| Permcath |  | 0.074 | 0.179 | -0.277 | 0.424 | 0.680 | 0.099 | 0.070 | 2.961 | 0.209 | 5.78 |
| Frequency of dialysis (n/week) | 413 | -0.249 | 0.269 | -0.776 | 0.278 | 0.355 | 0.108 | 0.074 | 2.952 | 0.208 | 6.36 |
| Dialysis session duration (hours) | 413 | 0.060 | 0.246 | -0.422 | 0.543 | 0.806 | 0.096 | 0.069 | 2.963 | 0.209 | 5.51 |
| eKt/V | 371 | -0.081 | 0.288 | -0.645 | 0.484 | 0.780 | 0.074 | 0.065 | 3.157 | 0.235 | 3.02 |
| Serum albumin (g/L) | 408 | -0.004 | 0.019 | -0.042 | 0.034 | 0.837 | 0.103 | 0.071 | 2.945 | 0.209 | 6.15 |
| Urea (mmol/L) | 413 | -0.008 | 0.007 | -0.021 | 0.005 | 0.246 | 0.092 | 0.067 | 2.956 | 0.208 | 5.31 |
| Residual Clearance (%) | 32 | 0.007 | 0.053 | -0.098 | 0.111 | 0.897 | 0.000 | 0.000 | 3.935 | 0.984 | 0.00 |
| CRP (mg/L) | 320 | 0.001 | 0.004 | -0.006 | 0.008 | 0.803 | 0.084 | 0.075 | 3.102 | 0.249 | 2.77 |
| Hemoglobin (g/L) | 413 | -0.008 | 0.007 | -0.021 | 0.006 | 0.261 | 0.098 | 0.070 | 2.954 | 0.208 | 5.73 |
| Phosphorus (mmol/L) | 411 | 0.316 | 0.208 | -0.091 | 0.723 | 0.128 | 0.113 | 0.075 | 2.949 | 0.209 | 7.04 |
| Calcium (mmol/L) | 412 | 0.548 | 0.542 | -0.514 | 1.610 | 0.312 | 0.092 | 0.067 | 2.964 | 0.209 | 5.09 |
| Potassium (mmol/L) | 413 | 0.021 | 0.129 | -0.232 | 0.274 | 0.869 | 0.099 | 0.071 | 2.962 | 0.209 | 5.65 |
| y-GT (U/L) | 370 | 0.002 | 0.001 | -0.001 | 0.004 | 0.161 | 0.115 | 0.081 | 2.783 | 0.208 | 5.84 |
| Total Bilirubin µmol/L | 139 | -0.010 | 0.019 | -0.047 | 0.026 | 0.571 | 0.337 | 0.234 | 2.544 | 0.312 | 11.44 |
| Ferritin (µg/L) | 403 | 0.000 | 0.000 | 0.000 | 0.001 | 0.125 | 0.097 | 0.070 | 2.958 | 0.211 | 5.40 |
| PTH (pmol/L) | 398 | 0.003 | 0.004 | -0.004 | 0.010 | 0.443 | 0.095 | 0.070 | 3.020 | 0.217 | 5.11 |
| Filter composition (reference: AN69) | 413 |  |  |  |  |  |  |  |  |  |  |
| Modified cellulose |  | -0.577 | 1.768 | -4.041 | 2.887 | 0.744 | 0.111 | 0.076 | 2.940 | 0.208 | 6.5 |
| MCO |  | 0.893 | 0.867 | -0.808 | 2.593 | 0.303 |  |  |  |  |  |
| Polyethersulfone |  | 0.215 | 0.496 | -0.758 | 1.187 | 0.666 |  |  |  |  |  |
| Polysulfone |  | 0.349 | 0.390 | -0.415 | 1.114 | 0.370 |  |  |  |  |  |
| Triacetate |  | 0.683 | 0.583 | -0.460 | 1.826 | 0.242 |  |  |  |  |  |

* Significance at p ≤ 0.05
